# Multiple components of environmental change drive populations of breeding waders in seminatural grasslands

**DOI:** 10.1002/ece3.4514

**Published:** 2018-09-27

**Authors:** Karsten Laursen, Javier Balbontín, Ole Thorup, Henrik Haaning Nielsen, Tommy Asferg, Anders Pape Møller

**Affiliations:** ^1^ Department of Bioscience Aarhus University Rønde Denmark; ^2^ Departamento de Zoología Facultad de Biología Sevilla Spain; ^3^ Amphi Consult Ribe Denmark; ^4^ Avifauna Consult Vesløs Denmark; ^5^ Ecologie Systématique Evolution CNRS Université Paris‐Sud AgroParisTech, Université Paris‐Saclay Orsay France

**Keywords:** climate change, environmental change, fertilizer, land use, long‐term studies, nutrients, precipitation, study methods, temperature

## Abstract

Environments are rapidly changing due to climate change, land use, intensive agriculture, and the impact of hunting on predator populations. Here, we analyzed long‐term data recorded during 1928–2014 on the size of breeding populations of waders at two large nature reserves in Denmark, Vejlerne and Tipperne, to determine the effects of components of environmental change on breeding populations of waders. Environmental variables and counts of waders were temporally autocorrelated, and we used generalized least square (GLS) by incorporating the first‐order autoregressive correlation structure in the analyses. We attempted to predict the abundance of waders for short‐term trends for two nature reserves (35 years) and for long‐term trends for one nature reserve (86 years), using precipitation, temperature, nutrients, abundance of foxes *Vulpes vulpes*, area grazed, and number of cattle. There was evidence of impacts of nutrients, climate (long‐term changes in temperature and precipitation), grazing, mowing, and predation on bird populations. We used standard random effects meta‐analyses weighted by (*N*–3) to quantify these mean effects. There was no significant difference in effect size among species, while mean effect size differed consistently among environmental factors, and the interaction between effect size for species and environmental factors was also significant. Thus, environmental factors affected the different species differently. Mean effect size was the largest at +0.20 for rain, +0.11 for temperature, −0.09 for fox abundance, and −0.03 for number of cattle, while there was no significant mean effect for fertilizer, area grazed, and year. Effect sizes for two short‐term time series from Tipperne and Vejlerne were positively correlated as were effect sizes for short‐term and long‐term time series at Tipperne. This implies that environmental factors had consistent effects across large temporal and spatial scales.

## 1 INTRODUCTION

Breeding populations of waders are declining across western Europe (Thorup, 2006; Roodbergen, van der Wert, & Hötker, 2012; Robinson, Morrison, & Baillie, 2014). The reasons for these declines include intensified agricultural practice, reclamation of coastal habitats, increasing predation pressure, human disturbance, and climate change (Beintema & Müskens, 1987; Wilson, Ausden, & Milsom, 2004; Smart, Gill, Sutherland, & Watkinson, 2006; Holm & Laursen, 2009; Roodbergen et al., 2012; Stephens et al., 2016). Demographic studies show that the declines are mainly caused by poor chick survival rather than adult survival (Roodbergen, Klok, & Schekkerman, 2008; Roodbergen et al., 2012). Low breeding success is caused by losses of nests to predation and flooding (Hötker & Segebade, 2000; Van de Pol et al., 2010; Bellebaum & Bock, 2009; Thorup & Koffijberg, 2016). Studies of wader populations during recent years have focused on farmland revealing complex interactions between agricultural practice and climate, affecting the survival of young (Kleijn et al., 2010; Schroeder et al., 2012). Young waders have to find their own food, and due to high energy requirements and poor abilities to save energy, they operate within narrow energetic margins, for example, a balance between quantity and quality of food (Schekkerman & Visser, 2001; Maier, 2013). To this array of parameters influencing wader populations, we included change in nutrient load in the environment as a proxy for primary productivity and amount of benthos and thus indirectly carrying capacity for breeding birds (Philippart et al., 2007; Møller, Flensted‐Jensen, Laursen, & Mardal, 2015). Thus, nutrient load can be an important parameter, although a straightforward relationship between nutrients and amount of invertebrate food for young cannot be expected (Schekkerman & Beintema, 2007). Outside the breeding season during migration and at wintering sites, several wader species stage and forage in marine estuaries and along coasts that are influenced by nutrients (Vitousek, Mooney, Lubchenko, & Melillo, 1997; Windolf, Blicher‐Mathiesen, Carstensen, & Krovang, 2012).

Different components of environmental change such as climate change, land use, and fisheries are currently being described as determinants of poor reproductive performance, reduced survivorship, and declining population trends for waders and other bird populations (Frederiksen, Wanless, Harries, Rothery, & Wilson, 2004; Schroeder et al., 2012; Maier, 2013). However, few studies have demonstrated for climate change that demographic variables determine population size (Dunn & Møller, 2014; Robinson et al., 2014; Stephens et al., 2016). Attempts to make integrated analyses of the relative contributions of multiple factors accounting for such trends are scarce. A number of studies have recently investigated the effects of climate change and land use on population size (Møller, Flensted‐Jensen, & Mardal, 2007; Pimm, 2009; Eglington & Pearce‐Higgins, 2012; Mantyka‐Pringle, Martin, & Rhodes, 2012; Jørgensen et al., 2015; Martin, van Dyck, Dendoncker, & Titeux, 2013; Møller & Laursen, 2015). This leaves a number of additional factors in need of study, including population changes and effects of industrialized agriculture with high levels of fertilizer use. Since such analyses quickly include many predictors, they are resource demanding and only feasible when based on long‐term studies. Unfortunately, most time series in ecological research are short and only rarely exceed 50 years, thereby often preventing inclusion of all or even most crucial predictors and certainly not inclusion of interactions among variables.

The aims of this study were (a) to quantify the effect size for the impact of environmental conditions on the population size of seven wader species; (b) to analyze the effects of climate, nutrients, land use, and predator abundance on size of local breeding populations in a 86‐year time series of wader bird communities at Tipperne and two short‐term time series of 35 years at Tipperne and Vejlerne; (c) to test for consistency in effect size between one short‐term and one long‐term time series at Tipperne and for one short‐term time series of effect sizes at Tipperne and one short‐term time series at Vejlerne. We did so by analyzing the breeding abundance of seven wader species during 1928–2014 at the nature reserve Tipperne, Denmark, and during 1978–2014 at the nature reserve Vejlerne, Denmark.

## 2 METHODS

### 2.1 Sites, wader species, and study periods

**Figure 1:**
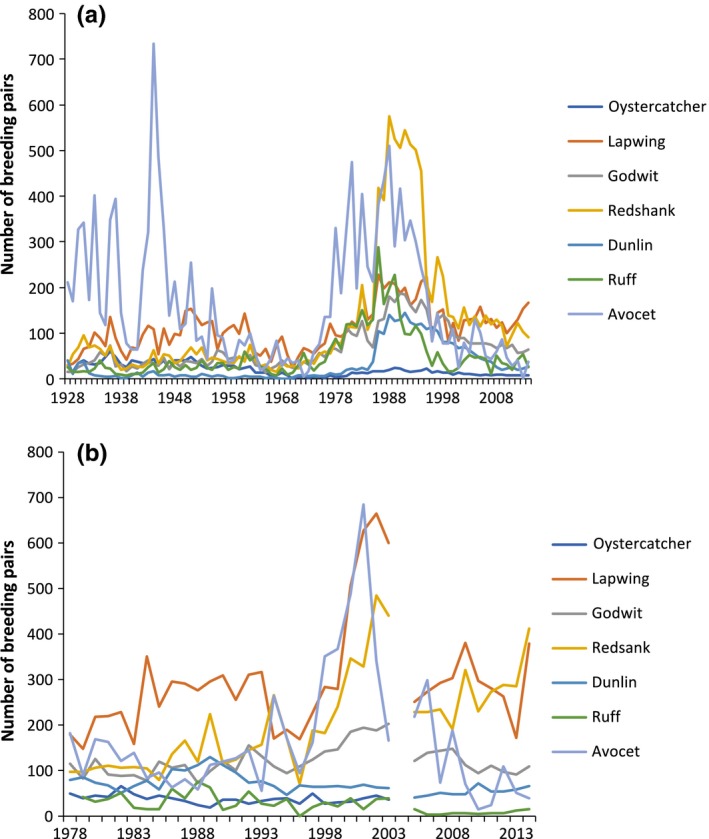
Number of breeding pairs of seven wader species at (a) Tipperne during 1928–2014 and (b) Vejlerne during 1978–2014

We studied breeding waders at two seminatural grassland sites (Tipperne and Vejlerne) in western Denmark extensively farmed, for example, grazed by cattle at low density, late mowing, and without direct use of fertilizer (see site description in Supporting information Appendix S1). We focused on the wader bird community composed of seven species: Oystercatcher *Haematopus ostralegus*, lapwing *Vanellus vanellus*, black‐tailed godwit *Limosa limosa*, redshank *Tringa totanus*, dunlin *Calidris alpina*, ruff *Philomachus pugnax,* and avocet *Recurvirostra avocetta*. Data on bird numbers are available at Tipperne during 1928–2014 and at Vejlerne 1978–2014, except for 2004 and for oystercatcher during 2004–2014 (see Fig. 1). They are all migratory birds with the early breeding species arriving in March (oystercatcher, lapwing), while the other species arrive in April. The two study areas are NATURA‐2000 sites that are strongholds for breeding waders in Denmark (Thorup, 2004).

### 2.2 Census methods

Census methods are described by Møller (1983), Thorup (1998), and Kjeldsen (2008). They have been adjusted during the study period due to change in vegetation height. At Tipperne during 1928–1957, nests were searched intensively. During 1958–1964, nest searches were supplemented by mapping birds giving alarm calls to identify territories. In 1965–1985, breeding birds giving alarm calls were mapped together with nests. From 1986, breeding waders were mapped from a distance with a telescope (60× magnification) supplemented with mapping events of warning birds. These methods were grouped in three categories and entered in the statistical analyses as a factor with the following levels: nest search, nest search and territory mapping, and territory mapping and telescope use. A detailed description of the census methods is given in Supporting information Appendix S1.

### 2.3 Climate

Climate estimated as long‐term change in precipitation and temperature together with groundwater level is known to affect breeding wader populations (Thorup, 1998; Bellebaum & Bock, 2009; Schroeder et al., 2012; Maier, 2013). We used mean of daily maximum air temperature (°C) in April and sum of monthly precipitation for March, April, and May (mm, data from Danish Meteorological Institute). We used data on climate measured at a local coastal weather station at Vestervig. Groundwater at Tipperne and water level at the outlet sluice at Vejlerne were measured, but not included in the environmental analysis due to significant correlations (*p* < 0.05) with precipitation in most spring months.

### 2.4 Nutrients

Data on fertilizer from farmland in Denmark were estimated for 1928–2014 as the annual amount of outlet of total‐N (tons) to coastal waters (Conley et al., 2007) and updates by Hansen (2015) and Thodsen et al. (2016). Each update was calibrated to the former level. This data set was used for the long‐term statistical analyses for Tipperne. From 1989, data on nitrogen (μg/L) concentration were collected in Ringkøbing Fjord (surrounding Tipperne) and for nitrogen in Limfjorden (adjacent to Vejlerne) as a part of the national monitoring program NOVANA (data from Environment Centre Ringkøbing; both sites; Hansen, 2015). These data sets were used for the short‐term statistical analyses at Tipperne and Vejlerne.

### 2.5 Management and predation

Habitat management as grazing and mowing is important for maintaining populations of breeding waders while predators reduce breeding success (Thorup, 1998; Bellebaum & Bock, 2009; Maier, 2013). At Tipperne, we have data on the number of cattle, area grazed (ha), and the relative area mowed since 1931. Due to qualitative information on area mowed, it was classified as 0 (0%–5% of the meadows mowed), 1 (6%–25% mowed), 2 (26%–50% mowed), or 3 (51%–75% mowed). At Vejlerne, data on the number of cattle, the area grazed, and the area mowed were available.

**Figure 2:**
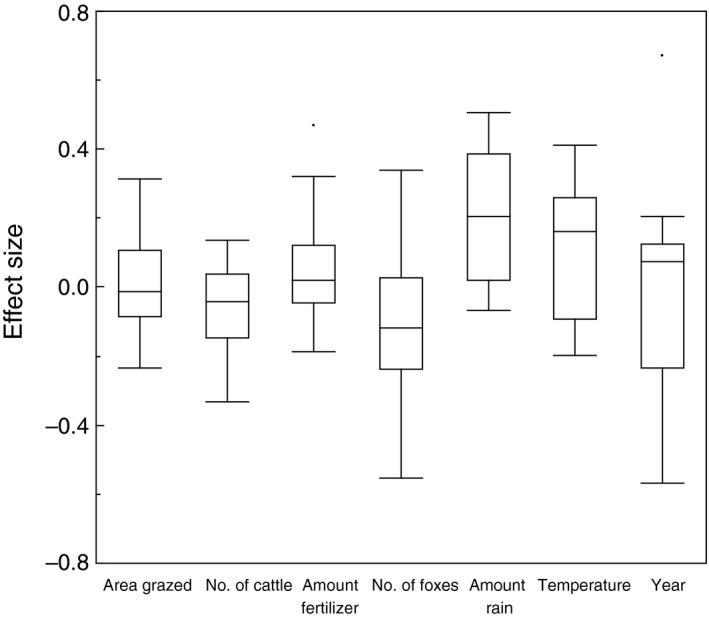
Box plots of effect size for impacts of different environmental conditions on population size and population trends of waders. The box plots show medians, quartiles, 5 and 95 percentiles, and extreme values

We analyzed the effects of foxes on nest predation (Thorup, 1998; Kjeldsen, 2008). Information about fox abundance was taken from the official Danish bag statistics, which goes back to 1941. From this year up to 1972, we used the national bag size of foxes as a proxy for fox abundance (annual number of foxes shot in Denmark). From 1973 to 2014, information exists about fox bag at the country level (annual number of foxes shot per county surrounding Tipperne and Vejlerne), and we used this local information to account for differences between regions. Environmental data for Tipperne and Vejlerne are shown in Supporting information Tables S1 and S2.

### 2.6 Statistical methods

We used generalized least square (GLS) that allows errors to be correlated and to have unequal variances. We tested for temporal autocorrelation for each wader species building two GLS, one of them incorporating the first‐order autoregressive correlation structure for year and the other one without it. We compared both models using ANOVA and likelihood‐ratio test (LRT) to evaluate which model performed better (Pinheiro & Bates, 2000). All analyses showed evidence of temporal autocorrelation (all *p *< 0.01). Hence, we used GLS that incorporate the first‐order autoregressive correlation structure for year to evaluate for each wader species seven predictors explaining variation in population size across years. The predictors were temperature in April, total precipitation from February to May, amount of nutrients, number of cattle, total area grazed, number of foxes, and year. Count numbers for each wader species were log‐transformed prior to analyses (except for lapwing counts that showed a distribution close to a Gaussian curve). All predictors were standardized to a mean of zero and *SD *= 1 to ensure that all variables being at the same scale and the intercept being interpretable. Residuals of each model were visually inspected for deviation for normality using normal QQ plots, and heteroskedasticity was also visually inspected with plots of residuals against the fitted values. We built models for long‐term and short‐term data at Tipperne and short‐term data at Vejlerne. We employed the libraries “nlme” (Pinheiro, Bates, DebRoy, & Sarkar, 2018) and used R version 3.3.3 (R Core Team 2017).

We estimated effect sizes as Pearson's product–moment correlation coefficients by using standard conversions (Rosenthal, 1994). These effect size analyses only included few observations, implying that the statistical power of any specific analysis is low. We adopted Cohen's (1988) recommendations for the magnitude of effects being small (Pearson *r* = 0.10, explaining 1% of the variance), intermediate (*r* = 0.30, explaining 9% of the variance), or large (*r* = 0.50, explaining 25% of the variance).

## 3 RESULTS

### 3.1 Effect size, environmental change, and species at Tipperne during 1928–2014

Overall effect size weighted by (*N*–3) was on average +0.033 (*SE *= 0.016), *N *= 147, marginally differing from zero (*t *= 2.02, *df *= 146, *p *= 0.045). There was no significant difference in effect size between the two sites (*F *= 0.254, *df *= 1, 145, *r* ^2^ **= 0.00, *p *= 0.615 (Figure 2)).

Effect size did not differ significantly among species (*F *= 1.08, *df *= 6, 140, *r* ^2^ **= 0.0003, *p *= 0.377). The four largest effect sizes were for foxes reducing the short‐term abundance of avocet at Vejlerne (*r *= −0.552, *t *= 2.90, *df *= 26, *p *= 0.0074), rain increasing the long‐term abundance of lapwing at Tipperne (*r *= +0.427, *t *= 3.58, *df *= 66, *p *= 0.0077), temperature increasing the long‐term abundance of godwit at Tipperne (*r *= +0.412, *t *= 3.44, *df *= 66, *p *= 0.001), and foxes reducing the short‐term abundance of lapwing at Vejlerne (*r *= −0.339, *t *= 3.42, *df *= 26, *p *= 0.0021). Short‐term effect size for godwit at Vejlerne increased with nutrients with a large effect of +0.468, *t *= 2.476, *df *= 26, *p *= 0.020, and avocet in the long term at Tipperne likewise had a strong positive effect size of +0.313, *t *= 2.655, *df *= 66, *p *= 0.0099.

**Figure 3:**
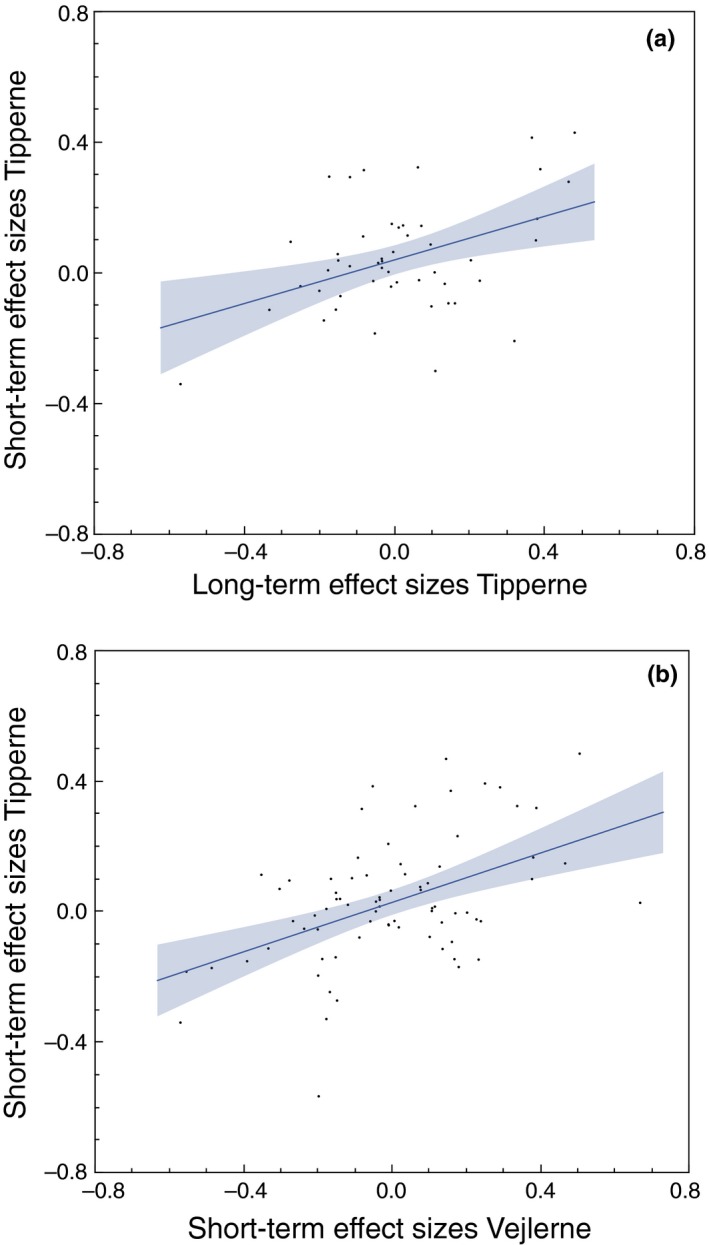
(a) Relationship between effect sizes for different characters between short‐term studies at Tipperne and long‐term studies at Tipperne. The lines show the positive linear regressions. (b) Relationship between effect sizes for different characters between short‐term studies at Tipperne and short‐term studies at Vejlerne

Effect size differed significantly among environmental variables (Figure 3; *F *= 5.797, *df *= 6, 140, *r* ^2^ **= 0.16, *p* < 0.0001). The interaction between species and environmental variables was also statistically significant (Figure 3; *F *= 3.415, *df *= 36, 140, *r* ^2^ **= 0.44, *p* < 0.0001). Thus, not all six environmental variables had similar effects on the different species. Precipitation had an intermediate significant positive effect on abundance, while temperature had a significant positive effect, foxes a significant negative effect, number of cattle a significant negative effect, and area grazed a significant positive effect (Figure 3; Table 1).

**Table 1:** Mean (*SE*) effect sizes for environmental factors at Tipperne and Vejlerne and tests for difference from the null hypothesis of zero in Wilcoxon matched‐pairs signed‐rank tests

Variable	Mean	*SE*	Wilcoxon W	*N*	*p*
Year	−0.014	0.053	−9,330	21	0.13
Amount of fertilizer	+0.024	0.033	4,075	21	0.51
Area grazed	+0.031	0.030	33,871	21	<0.0001
No. of cattle	−0.032	0.026	−47,365	21	<0.0001
No. of foxes	−0.086	0.041	−76,427	21	<0.0001
Temperature	+0.110	0.044	82,291	21	<0.0001
Precipitation	+0.197	0.040	19,652	21	<0.0001

### 3.2 Correlations between effect sizes at different sites and in periods of different duration

For the data from Tipperne, there was a positive correlation between short‐ and long‐term effect sizes (Figure 3a; *F *= 10.187, *df *= 1, 47, *r* ^2^ **= 0.16, *p *= 0.0025, estimate (*SE*)* *= 0.334 (0.105), effect size +0.42). Thus, population changes have been parallel at short and at long terms. Similarly, there was a positive correlation between short‐term effect size at Tipperne and short‐term effects at Vejlerne (Figure 3b; *F *= 12.260, *df *= 1, 47, *r* ^2^ **= 0.19, *p *= 0.0010, estimate (*SE*)* *= 0.391 (0.112), effect size +0.45). Therefore, specific environmental factors had similar effects across spatial scales.

## 4 DISCUSSION

The main findings of this long‐term study of population trends of breeding waders at two nature reserves in Denmark since 1928 and 1978 were significant impacts of multiple components of environmental change. In the present study, we tested for consistency in effect sizes for environmental variables hypothesized to affect population trends of waders. We focused on a suite of environmental change parameters hypothesized to act on breeding wader populations, although these species are migrants that spend the nonbreeding season along the East Atlantic Flyway from the Wadden Sea in the north to West Africa in the south (Bønløkke et al., 2006). The long‐term time series dating back to 1928 allowed us to test for heterogeneity in strength of the relationship between abundance of breeding waders and multiple components of environmental change. In particular, we were able to rank environmental components in terms of importance. We found no significant difference among species in effect size, but we documented significant heterogeneity among environmental factors, and these effects varied among species. Importantly, effect sizes for the two short time series at Tipperne and Vejlerne were positively correlated, and that was also the case when relating effect sizes for the short term and the long term at Tipperne. Thus, effect sizes for environmental factors were consistent across temporal and spatial scales.

Climate change is currently considered a major determinant of phenology, demography, distribution, and population trends (reviews in Møller, Fiedler, & Berthold, 2010; Pearce‐Higgins & Green, 2014; Stephens et al., 2016). However, there is only little empirical evidence suggesting that population size of birds is impacted by climate change (Dunn & Møller, 2014; Stephens et al., 2016). Arrival date and early breeding in waders are clearly affected by warmer temperature in spring, although altered by agricultural practice (Petersen, Meltofte, & Tøttrup, 2012; Schroeder et al., 2012). We showed considerable fluctuations in breeding populations of waders at Tipperne since 1928, explained by spring temperature and in particular precipitation. We documented a mean effect size of +0.197 (*SE *= 0.040) for spring precipitation and a weaker effect size of +0.110 (0.044) for spring temperature. Since both temperature and precipitation have increased, this implies that the changes in climate are those that improved the abundance of breeding waders the most. Temperature and precipitation are known to affect reproduction in waders (Schroeder et al., 2012), and populations responding positively to climate change are likely to increase (Møller, Rubolini, & Lehikoinen, 2008; Stephens et al., 2016).

Land use has changed consistently in the two study sites due to management. Interestingly, we had detailed information on the area grazed and therefore on its relative importance for breeding waders. Grazing reduced the height of the vegetation, slowed down or even reversed successional processes, and increased arthropod biomass, which in turn had beneficial effects on growth of young (Norris et al., 1998; Eglington et al., 2008; Maier, 2013). In addition, vegetation height affects the ability of waders to observe predators at a distance, but also prevents use of suitable nesting sites and access to preferred foraging habitats for young due to tall swards (Cramp, 1985; Kleijn et al., 2010). We found evidence of a weak mean effect of the number of cattle on population size of different species of waders at the two study sites. However, the weak mean effect of number of cattle of −0.032 (0.026) implies that this management tool in fact had a detrimental effect rather than a beneficial effect on the number of breeding waders. In contrast, the area grazed had a mean effect of +0.031 (0.030). This implies that the number of breeding waders improved marginally with the area grazed by cattle.

We analyzed the effects of foxes on population size of breeding waders that mainly act through effects on nest predation and to a smaller extent on predation on adult birds (Beintema & Müskens, 1987; Meisner et al., 2014). We expected that foxes would negatively impact population trends of waders. Indeed, we found an expected mean effect of −0.106 (0.050). A total of 16 out of 21 effect sizes were negative. Differences in effects of fox predation over time and between sites could be the consequence of outbreaks of the sarcoptic mange disease caused by the skin‐dwelling mite *Sarcoptes scabiei*. Scabies can reduce fox populations locally and nationally with effects on the breeding waterbird community (Forchhammer & Asferg, 2000; Clausen & Kahlert, 2010). There was a negative impact of foxes on waders, as shown by Roodbergen et al. (2012).

Fertilizer increases nutrient availability, primary productivity, and subsequent effects on the abundance of animals at higher trophic levels in the marine environment (Phillippart et al., 2007; Møller et al., 2015). Mean effect size of fertilizer on waders was only +0.024 (0.033), which was not significantly different from zero. Here, we documented two relatively large effects. We found that effect size for godwit at Vejlerne increased with fertilizer use with a large effect size of +0.468, *t *= 2.476, *df *= 26, *p *= 0.020, and avocet in the long‐term at Tipperne likewise had a strong positive effect size of +0.313, *t *= 2.655, *df *= 66, *p *= 0.0099. Tipperne and the surrounding Ringkøbing Fjord are strongly impacted by drainage from Skjern River, which transports huge amounts of nutrients from large surrounding agricultural areas (Petersen et al., 2008).

This study has significant future prospects. First, we only analyzed the main factors that most likely affect populations and population trends of breeding waders. Clearly, it would be interesting to assess to which extent interactions between factors may impact populations and population trends. Second, this study has important implications for conservation in terms of management of nature reserves, management of populations of predators such as foxes, and management of vegetation as affected by grazing. Clearly, most nature reserves, often small in size, will be impacted by a number of factors related to environmental change, but also influenced by surrounding areas that are much larger. We have shown here that effect sizes for short‐ and long‐term effects are positively correlated and that different factors affect the size of breeding wader populations.

In conclusion, we have analyzed unique long‐term data on breeding populations of waders with the longest time series starting in 1928. When analyzing the relationship between the abundance of breeding waders and multiple components of environmental change, we found significant evidence of effects. While there were positive correlations between effect sizes for short‐term studies at different sites, or long‐ and short‐term effect sizes at the same site, these correlations were relatively strong accounting for 10%–15% of the variance. These findings emphasize the importance of considering heterogeneity in future monitoring schemes.

## AUTHORS CONTRIBUTION

APM conceived the project; OT and HHN collected the data; APM and KL analyzed the data; KL, APM, OT, HHN, and TA wrote the manuscript.

## DATA ACCESSIBILITY

We suggest to store the data at TreeBASE, if the paper is accepted.
