# Supplementary material for: Multiple components of environmental change drive populations of breeding waders in seminatural grasslands
Source: Ecol Evol. 2018 Sep 27;8(21):10489–96. doi: 10.1002/ece3.4514 (PMC6238131; doi:10.1002/ece3.4514)
Supplement: Supplementary file 1 [file ECE3-8-10489-s001.docx]

**Electronic Supporting Information, Appendix S1**

**Site descriptions**

Tipperne is a nature reserve situated at a peninsula in the southern part of Ringkøbing Fjord, Western Denmark, near the North Sea coast. The reserve covers 700 ha dominated by natural grassland and reed beds. It is surrounded by large areas with shallow brackish water. The vegetation was grazed and mowed intensively during 1928-1945. Thereafter, management was reduced gradually and the vegetation became dominated by communities of tall plant species as reed *Phragmites australis*. During the 1970’s grazing was re-introduced and in the 1980’s supplemented withß mowing during late summer (Thorup 1998). The central and dryer parts of the reserve were mowed later. The wetter parts were mowed occasionally when the conditions allowed use of machinery. Mowing took place in late July or early August, to prevent any effect on breeding waders. Grazing intensity during 1928-2014 was (mean (SE)) 0.75 (0.04) cattle per ha. Before 1931 the waterbody around Tipperne was almost fresh, but that year a canal was established from the North Sea to Ringkøbing Fjord and salinity increased gradually. Ringkøbing Fjord receives fresh water from a large catchment area via Skjern River. The river was straightened in the late 1960’s resulting in increased leaching of nutrients, eutrophication and sedimentation in Ringkøbing Fjord described as a regime shift in the ecosystem also influencing the water body surrounding Tipperne (Petersen et al. 2008). There is no tidal effect and flooding risk is small.

Vejlerne (2.620 ha) was formerly a shallow bay connected to Limfjorden that was reclaimed in 1868 and drained. However, farming stopped during the First World War and pumps were replaced with sluices leading water to Limfjorden. Later the habitats became dominated by wet grassland, lakes, reed beds and small canals (Kjeldsen 2008). A sluice situated in the southern part controls water level in the majority of the areas. Thus, there was no flooding risk. The grasslands were grazed by cattle to keep the vegetation short. The gazing intensity was (mean (SE) 0.5 (0.03) cattle per ha. However, the grazing regime changed in 1978-1990, when grazing intensity was low, and it peaked in 1991-1997 (Kjeldsen 2008). Parts of the meadows with high vegetation were mowed in late July or early August.

**Census methods of bird counts**

At Tipperne during 1928-1957 the vegetation was low and 2-3 persons searched intensively for wader nests during 2-4 weeks from May to mid-June (Thorup 1998). Incubating waders were also spotted from a 12 m high tower with a large telescope (maximum 110x magnification). During 1958-1964 the census methods were supplemented by mapping birds giving alarm calls to identify territories. In 1965-1972 the vegetation was high and more time was spent mapping breeding birds giving warning and alarm calls. During 1972-1985 the territories of breeding waders were mapped systematically by recording birds giving alarm calls, nests and chicks. From 1986 breeding waders were mapped from a distance with a telescope (60x magnification) from elevated points, supplemented with two mapping events (late April-mid May, mid May-mid June) of warning birds and birds with chicks (Thorup 1998). At Vejlerne (1978-2014) the territories of waders were mapped twice (late April-mid May and mid May-mid June, Kjeldsen 2008) from a distance by use of telescopes (60x magnification) and by mapping birds warning and giving alarm calls.
